# Integrated metagenomic and metaproteomic analyses reveal bacterial micro-ecological mechanisms in coral bleaching

**DOI:** 10.1128/msystems.00505-23

**Published:** 2023-10-26

**Authors:** Keke Cheng, Xinyang Li, Mengmeng Tong, Mui-Choo Jong, Zhonghua Cai, Huina Zheng, Baohua Xiao, Jin Zhou

**Affiliations:** 1 Shenzhen Public Platform for Screening and Application of Marine Microbial Resources, Shenzhen International Graduate School, Tsinghua University, Shenzhen, Guangdong, China; 2 Ocean College, Zhejiang University, Zhoushan, Zhejiang, China; 3 Institute of Environment and Ecology, Shenzhen International Graduate School, Tsinghua University, Shenzhen, Guangdong, China; 4 Shenzhen Institute of Guangdong Ocean University, Shenzhen, Guangdong, China; University of Guelph, Guelph, Canada

**Keywords:** coral bleaching, coral symbionts, coral-associated bacteria, metagenome, metaproteome

## Abstract

Coral bleaching has been rapidly increasing in recent years due to abnormally elevated temperature, leading to massive damage to coral reefs worldwide. Understanding the processes and micro-ecological mechanisms of coral symbionts in response to bleaching is crucial as evidence accumulates that micro-organisms (particularly the bacteria) contribute to the health and recovery of coral, especially during heat stress. However, the exact functional mechanism of bacteria has not yet been fully elucidated. In this study, we performed metagenomic and metaproteomic analyses of healthy and bleached *Acropora muricata* corals to identify taxonomic and functional shifts in coral symbionts during a natural thermal bleaching event on Hainan Island. The results showed that symbiont species tended to be more abundant in bleached corals than healthy corals, and the bacterial community appeared to be crucial to coral bleaching. The relative abundance of opportunistic pathogens dramatically increased in bleached corals, accompanied by the reduction of beneficial bacteria. In addition, a direct comparison of metagenomic data sets indicated major changes in functional genes, with bleached corals exhibiting significant metabolic enrichment, while healthy corals maintained lower metabolism and energy consumption. Carbohydrate-active enzyme genes were remarkably activated, and virulence factors were highly represented in bleached corals, which was directly related to the increased abundance of pathogenic bacteria. Metaproteomic analysis also demonstrated that bleaching greatly affected photosynthesis and energy metabolism of coral symbionts. Among them, the biological processes of photosynthesis and chlorophyll biosynthesis were common in healthy corals, while pathways involved in gluconeogenesis and apoptosis were significantly enriched in bleached corals. Bacteria-mediated processes in healthy corals contribute to maintaining the basic functions of symbionts and resisting stress, whereas the proliferation of pathogenic bacteria in bleached corals leads to metabolic abnormalities of symbionts, showing enhanced energy metabolism and catalytic activity. In summary, the two omics analyzes revealed that bleaching caused enormous physiological damage to corals, and bacterial imbalance and dysfunction were the potential micro-ecological mechanisms underlying this event.

**IMPORTANCE:**

Coral reefs worldwide are facing rapid decline due to coral bleaching. However, knowledge of the physiological characteristics and molecular mechanisms of coral symbionts respond to stress is scarce. Here, metagenomic and metaproteomic approaches were utilized to shed light on the changes in the composition and functions of coral symbiotic bacteria during coral bleaching. The results demonstrated that coral bleaching significantly affected the composition of symbionts, with bacterial communities dominating in bleached corals. Through differential analyses of gene and protein expression, it becomes evident that symbionts experience functional disturbances in response to heat stress. These disturbances result in abnormal energy metabolism, which could potentially compromise the health and resilience of the symbionts. Furthermore, our findings highlighted the highly diverse microbial communities of coral symbionts, with beneficial bacteria providing critical services to corals in stress responses and pathogenic bacteria driving coral bleaching. This study provides comprehensive insights into the complex response mechanisms of coral symbionts under heat stress from the micro-ecological perspective and offers fundamental data for future monitoring of coral health.

## INTRODUCTION

Coral reefs, known for their high biodiversity and economic value (1), have been facing alarming declines globally over the past few decades, largely due to coral bleaching triggered by rising seawater temperatures (2, 3). High temperatures also provide distinct advantages for coral pathogens, leading to a global increase in the prevalence of coral diseases (4). As the severity and frequency of coral bleaching and diseases increases, coral reef ecosystems are at risk of severe loss, emphasizing the importance of understanding coral resilience and acclimatization to environmental stressors.

The stress responses and fitness of corals are highly intricate due to their symbiotic relationship with diverse microbiota, comprising symbiotic algae, bacteria, fungi, archaea, protists, and viruses (5, 6). Coral hosts can benefit from these microbes through a range of biological processes which are critical to their metabolism and physiology (7). In particular, corals rely on endosymbiosis with photosynthetic algae of the family Symbiodiniaceae. Additionally, recent studies have revealed associations between corals and diverse bacteria that play a crucial role in coral health and viability (8, 9). When symbionts encounter adverse conditions, such as high temperature, the coral symbiosis may break down, which can be exacerbated by heat stress-induced pathogen invasion, ultimately leading to coral bleaching or death (10, 11). Consequently, understanding the physiological responses of coral microbial symbionts to heat stress is crucial for comprehending the response mechanisms of corals to bleaching and improving coral reef conservation efforts. However, the current knowledge on coral symbiont responses to environmental perturbations, such as ocean warming, remains relatively scant.

Recent studies have extensively investigated the associations of mass bleaching with coral-associated bacterial communities, revealing significant structural changes (12, 13). Bleaching was associated with a notable shift in the coral bacterial assemblages, with decreases in certain probiotic bacteria and colonization by potential opportunistic pathogens (14). Coral-associated bacterial communities appear to influence the capacity of the host to withstand heat stress, suggesting that altering the bacterial community may help corals to acclimate to harsh environments (15). Nevertheless, the molecular mechanisms of the bacterial stress responses that help to prevent coral bleaching remain largely unknown. Therefore, extensive functional studies are imperative to fully elucidate the associations of changes in bacterial communities with coral health and fitness.

The recent advancements and use of omics approaches have been instrumental to enhancing our understanding of coral microbial diversity and function, particularly regarding the metabolism and physiology of coral symbionts under environmental perturbations (16, 17). Metagenomic research has shown that the coral-associated microbiome provides the coral hosts with an increased number of gene functions involved in nutrient cycling (e.g., photosynthesis, phosphorus metabolism, and sulfur assimilation), along with genes involved in microbial activity, competition, and stress responses, which may contribute to the symbionts’ resilience to environmental changes and diseases (18). Genomic and proteomic analyses have shown that bacterial virulence and secondary metabolism genes were significantly enriched at high temperature (19, 20). Furthermore, a functional analysis of a coral metagenome revealed that environmental stressors increased the abundance of microbial genes involved in virulence, stress resistance, sulfur and nitrogen metabolism, motility and chemotaxis, and fatty acid and lipid utilization (21). Together, this information highlights the importance and complexity of the comprehensive responses of coral-associated bacteria to environmental stresses and enables the assessment of the functional characteristics of coral symbionts.

Currently, studies on coral microbial communities are burgeoning, while omics research on the micro-ecological mechanisms underlying coral symbionts’ responses to heat stress remains lacking. Given the devastating impacts of bleaching, more functional analyses are urgently needed to holistically understand the intricate mechanisms that lead to the collapse of coral symbiont communities. In 2020, corals near Hainan Island, South China, experienced unprecedented mass bleaching due to severe sea surface temperature anomalies (22). This event has provided us with a great opportunity to characterize the bacterial behavior (from structure to function) after coral bleaching in an *in situ* environment. We hypothesized that coral bleaching may be associated with disruption of the symbiotic microbial structure and alteration of the ratio of probiotics to potential pathogens, involving metabolic hyperactivity and disrupted symbiotic homeostasis. To test this hypothesis and determine the detailed factors associated with coral bleaching and the molecular mechanisms, we used metagenomic and metaproteomic analyses to investigate the genomic signatures and proteomic profiles of healthy and bleached corals near Hainan Island after the coral bleaching event. Our aims were to establish a baseline understanding of coral symbionts’ micro-ecological response mechanisms associated with coral bleaching and provide a useful reference for the health and recovery of coral reefs.

## RESULTS

### Differences in coral symbiont species between the healthy and bleached corals

**Fig 1:**
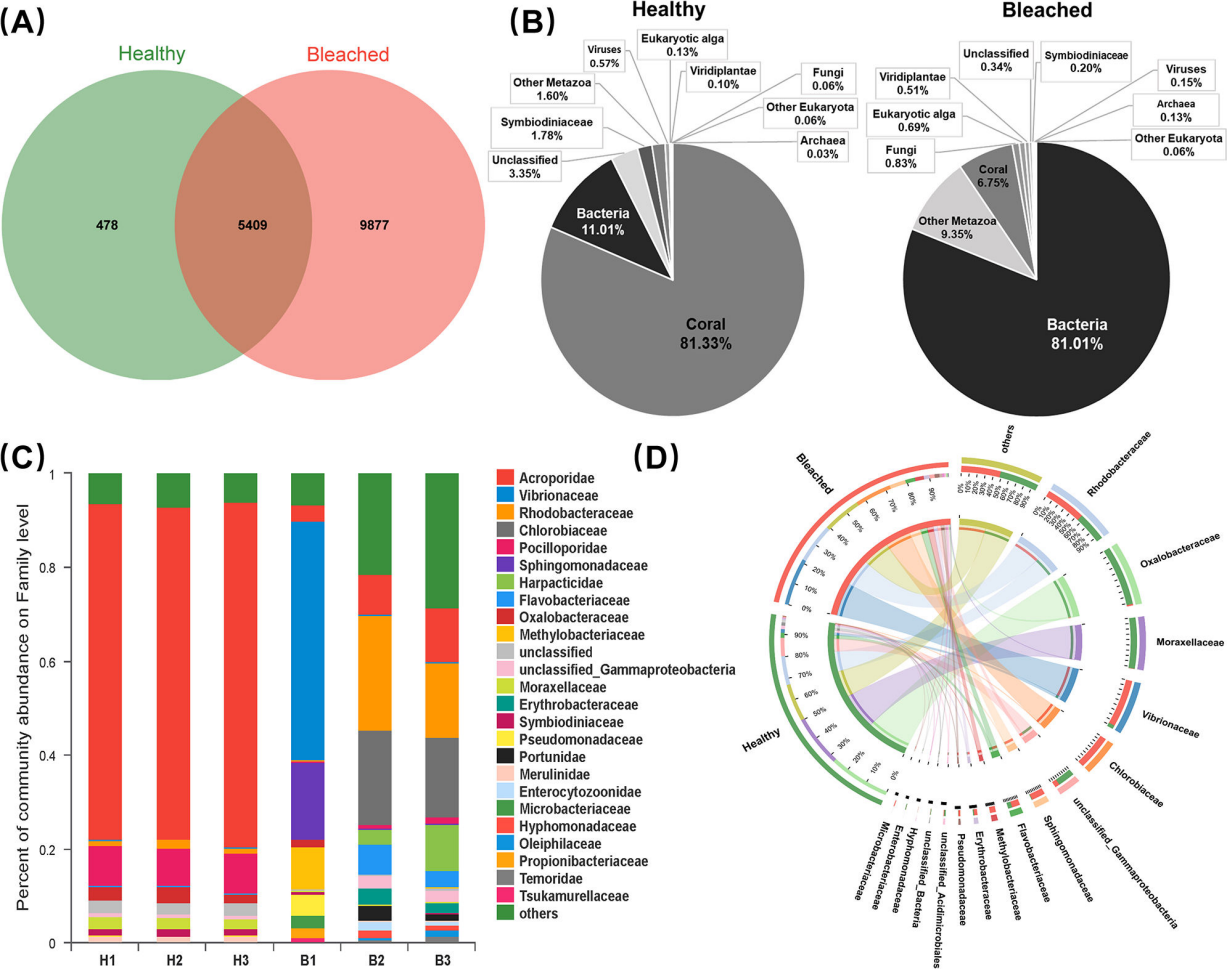
Species composition analysis of coral symbionts. (**A**) Venn diagram illustrating the number of symbiotic species in healthy and bleached corals. (**B**) Pie charts representing the percentage distribution of symbiotic species in healthy and bleached coral. (**C**) Relative abundances of symbiotic community at the family level in each coral individuals. (**D**) Relative abundance of bacterial distribution profiles at the family level in healthy and bleached coral.

DNA sequencing generated 460 million raw reads. After eliminating the low-quality sequences, there were 211,432,150 clean reads in the healthy library and 225,186,386 clean reads in the bleached library, with a mean of 70,477,383 [standard deviation (SD) = 8,116,148] and 75,062,129 (SD = 4,015,620) per replicate, respectively. Of the 15,764 annotated coral symbiont species (Fig. 1A), 478 were observed in the healthy group; 9,877 were observed in the bleached group; and 5,409 were shared by the two groups.

In the healthy group, 81.33% of the sequences were coral; 11.01% were bacteria; and 1.78% were Symbiodiniaceae (dinoflagellates) (Fig. 1B). Viruses, fungi, archaea, algae, and viridiplantae combined accounted for <1%. In the bleached group, bacteria were overwhelmingly dominant (81.01%), followed by metazoans (9.35%) and corals (6.75%) (Fig. 1B). The bleached group had significantly lower abundances of fungi (0.83%), eukaryotic algae (0.69%), and Viridiplantae (0.51%). Unlike the healthy group, Symbiodiniaceae accounted for only 0.2% in the bleached group. Regarding the family-level composition of coral symbionts, the healthy group was dominated by Acroporidae (71.94%) and Pocilloporidae (8.22%), both belonging to the class Anthozoa. In contrast, the bleached group was dominated by bacteria, including Vibrionaceae (17.06%), Rhodobacteraceae (13.54%), and Chlorobiaceae (12.33%) (Fig. 1C).

Bacteria were the most abundant organisms in the coral symbiont metagenome. Focusing on bacteria, the healthy and bleached groups were compared at the class (Fig. S2) and family (Fig. 1D) levels. Regarding bacteria at the class level, the top three in the two groups were Alphaproteobacteria, Gammaproteobacteria, and Chlorobia, accounting for >80% of the bacteria. Gammaproteobacteria (42.23%) and Betaproteobacteria (27.18%) were dominant in the healthy group, while Alphaproteobacteria (32.83%), Gammaproteobacteria (30.44%), and Chlorobia (22.28%) were dominant in the bleached group. Regarding bacteria at the family level, Oxalobacteraceae (27.45%) and Moraxellaceae (23.58%) were dominant in the healthy group, whereas Vibrionaceae (21.81%), Chlorobiaceae (16.34%), and Sphingomonadaceae (7.2%) were dominant in the bleached group. Rhodobacteraceae was dominant in both the healthy (13.25%) and bleached (17.95%) groups.

### Functional enrichment analyses of differentially abundant genes between the metagenomes of the healthy and bleached corals

**Fig 2:**
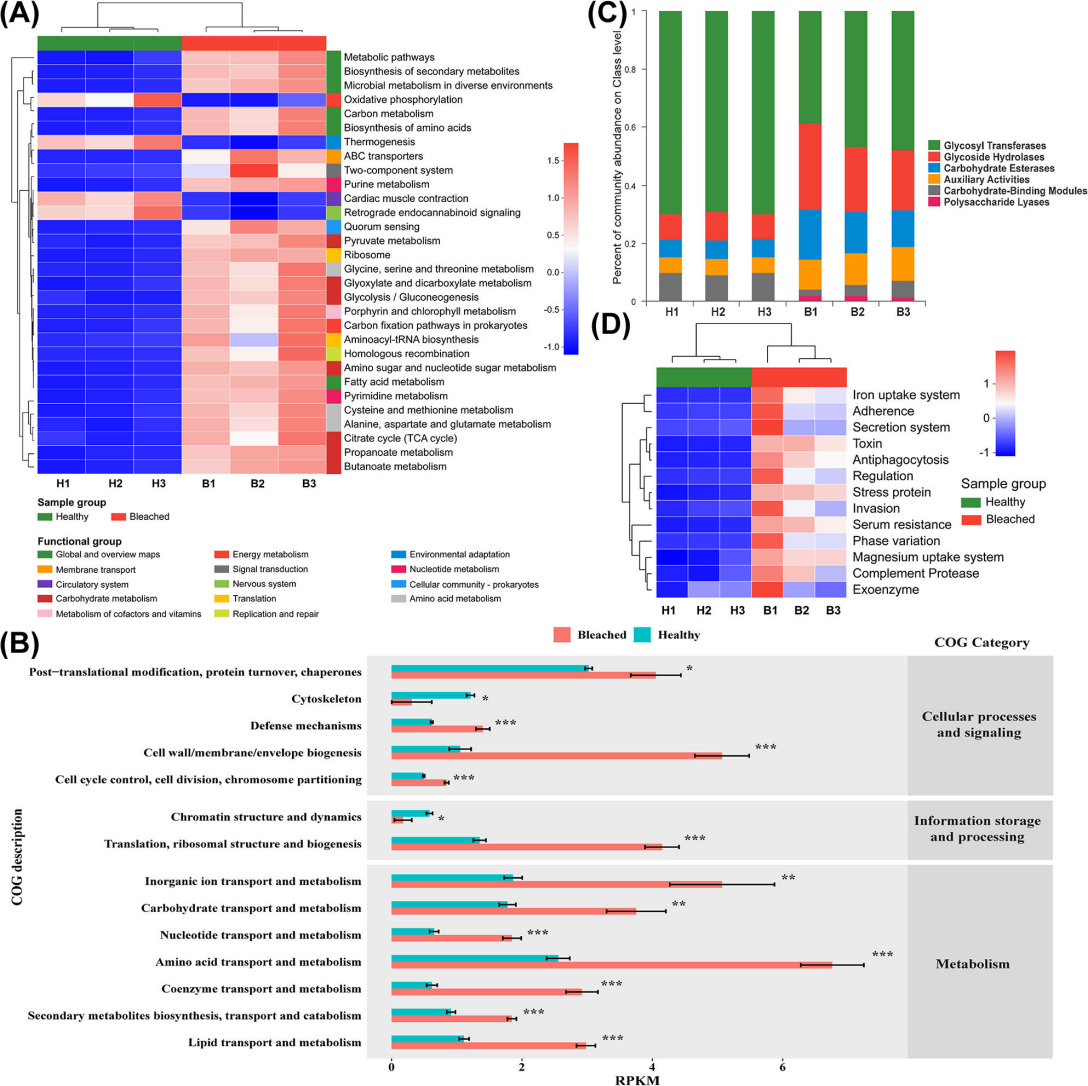
Metagenomic analysis of healthy and bleached corals. (**A**) Heatmap of hierarchical clustering analysis of the top 30 abundant KEGG functional categories (level 3) in healthy and bleached corals. (**B**) Cluster of Orthologous Groups of proteins (COG) analysis between healthy and bleached corals according to COG functional categories based on reads per kilobase per million mapped reads (RPKM). (**C**) Relative abundance variations of carbohydrate-active enzyme genes identified in each coral sample. (**D**) Cluster heatmap of virulence factor genes enriched in each coral individuals.

To assess the differences in physiological characteristics between the healthy and bleached groups, functional enrichment analyses were performed on the assembled metagenomes. The Kyoto Encyclopedia of Genes and Genomes (KEGG) analysis revealed distinct differences in the enrichment of major biochemical metabolic pathways between the bleached and healthy groups (Fig. 2A). Several metabolic categories were enriched in the bleached group, including metabolic pathways, biosynthesis of secondary metabolites, microbial metabolism in diverse environments, carbon metabolism, and biosynthesis of amino acids (*P* < 0.05). Moreover, metabolic pathways such as nucleotide metabolism, carbohydrate metabolism, amino acid metabolism, and cofactor and vitamin metabolism were significantly enriched in the bleached group (*P* < 0.05). Furthermore, pathways associated with membrane transport, signal transduction, replication and repair, and translation were much more enriched in the bleached group than in the healthy group (*P* < 0.05). Conversely, oxidative phosphorylation (energy metabolism subcategory), thermogenesis (environmental adaptation subcategory), circulatory system, and nervous system were enriched in the healthy group (*P* < 0.05).

The Cluster of Orthologous Groups of proteins (COG) analysis showed that most genes were enriched in the bleached group compared to the healthy group (*P* < 0.05), with the exception of cytoskeletal genes (involved in the COG category “Cellular processes and signaling”) and chromatin structure and dynamics genes (involved in the COG category “Information storage and processing”) (Fig. 2B). Notably, in the bleached group, genes related to protein modification and turnover, cell defense mechanism, cell membrane biosynthesis, cell cycle control, and ribosomal structure and biogenesis were enriched (*P* < 0.05). Additionally, regarding the COG category “metabolism,” genes involved in the transport and metabolism of inorganic ions, carbohydrates, nucleotides, amino acids, coenzymes, secondary metabolites, and lipids were also enriched in the bleached group (*P* < 0.05).

CAZyme genes encoded by coral-associated micro-organisms play a vital role in breaking down complex carbohydrates into components that can be absorbed by the host. Glycosyl transferases, which were the most common CAZyme genes, were higher in the healthy group (69.74%) than the bleached group (44.36%) (Fig. 2C). Carbohydrate-binding module genes were also higher in the healthy group (9.19%) than the bleached group (3.97%). In contrast, glycoside hydrolases (healthy group: 8.93%, bleached group: 24.23%), carbohydrate esterases (healthy group: 6.22%, bleached group: 14.75%), auxiliary activities (healthy group: 5.57%, bleached group: 11.03%), and polysaccharide lyases (healthy group: 0.34%, bleached group: 1.65%) were higher in the bleached group than the healthy group.

The Virulence Factor Database (VFDB) database was used to identify virulence factors in the healthy and bleached groups (Fig. 2D). Interestingly, the abundance of virulence factors was significantly higher in the bleached group than the healthy group (*P* < 0.05). These virulence factors were primarily related to ion uptake, such as iron and magnesium uptake, and invasion, including adherence, secretion, toxins, and regulation.

### Contribution of coral symbionts to functions

**Fig 3:**
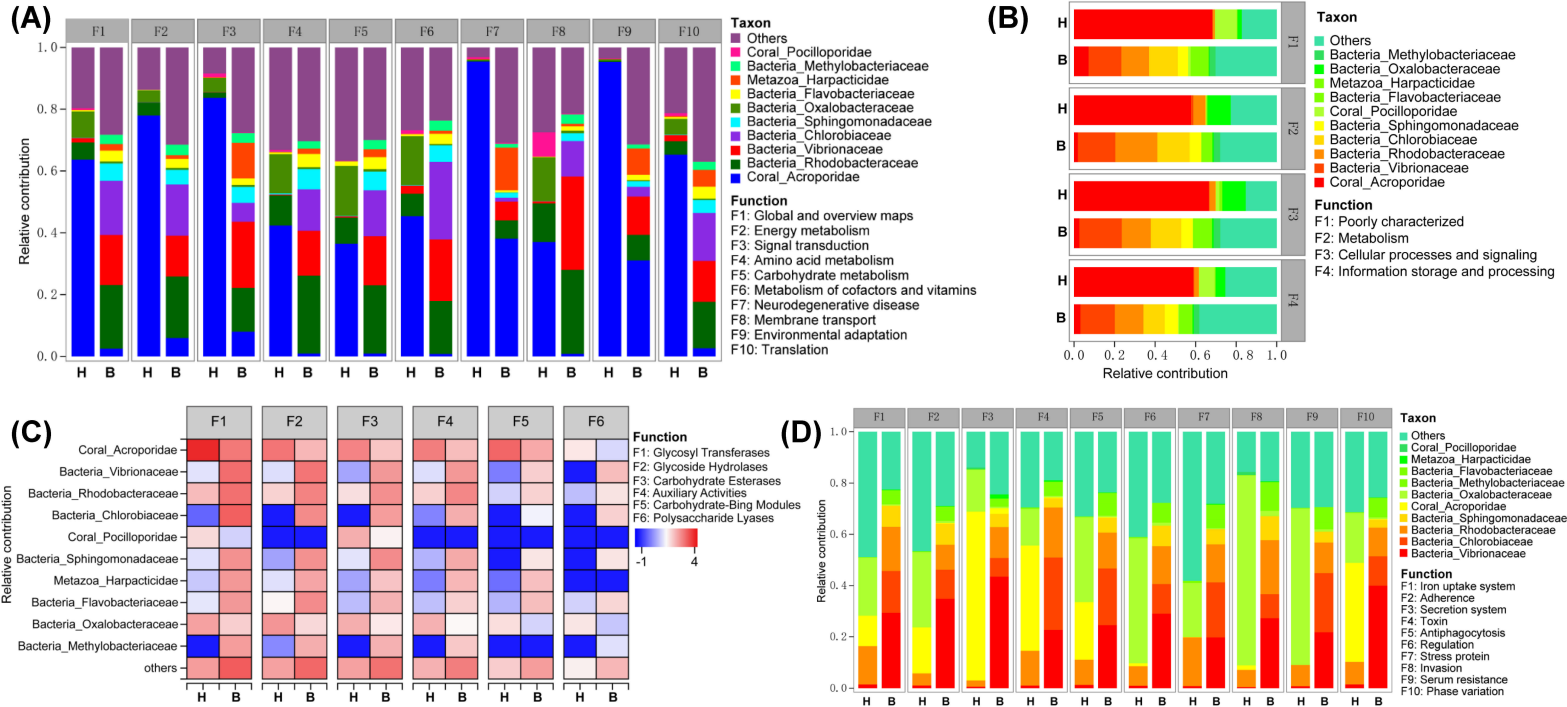
Species and functional contribution analysis. Family-level of species contribution to (**A**) KEGG functional categories (level 2), (**B**) COG functional categories, (**C**) carbohydrate-active enzyme genes, and (**D**) virulence factor genes.

To explore the relationships between the coral symbionts and functions, the contributions of the symbionts to the various functions were determined. Regarding the KEGG pathways enriched in the healthy group, the genes in the healthy group were mainly derived from the coral Acroporidae, which had a very high contribution to both signal transduction (83.72%) and environmental adaptation (95.36%) (Fig. 3A). Besides the coral host, the bacteria Rhodobacteraceae and Oxalobacteraceae contributed the most to the remaining functions in the healthy group. In the bleached group, the functions were mainly contributed by the bacteria Vibrioceae, Rhodobacteriaceae, and Chlorobacteriaceae. The contribution of coral Acroporidae to the various functions in the bleached group ranged from 0.83% to 8.0% for all functions except environmental adaptation, to which it contributed the most in the bleached group (31.07%).

Regarding the COG functions, the coral Acroporidae contributed to metabolism (57.71%), cellular processes and signals (66.56%), and information storage and processing (58.91%) in the healthy group, while the bacteria Vibrionaceae, Rhodobacteraceae, and Chlorobiaceae made important contributions in the bleached group (with relative contributions of the three bacteria combined ranging from 41.64% to 55.1%) (Fig. 3B).

Regarding the CAZymes, the coral Acroporidae made major contributions to glycosyl transferases, glycoside hydrolases, carbohydrate esterases, auxiliary activities, and carbohydrate-binding modules in the healthy group, whereas the bacteria Vibrionaceae, Rhodobacteraceae, and Chlorobiaceae made important contributions in the bleached group (Fig. 3C). Regarding the virulence factors, those in the healthy group were mainly contributed by the bacteria Rhodobacteriaceae and Oxalobacteraceae and the coral Acroporidae, whereas those in the bleached group were mainly contributed by the bacteria Vibrionaceae, Chlorobiaceae, and Rhodobacteraceae (Fig. 3D).

### Differential proteins between the healthy and bleached corals

**Fig 4:**
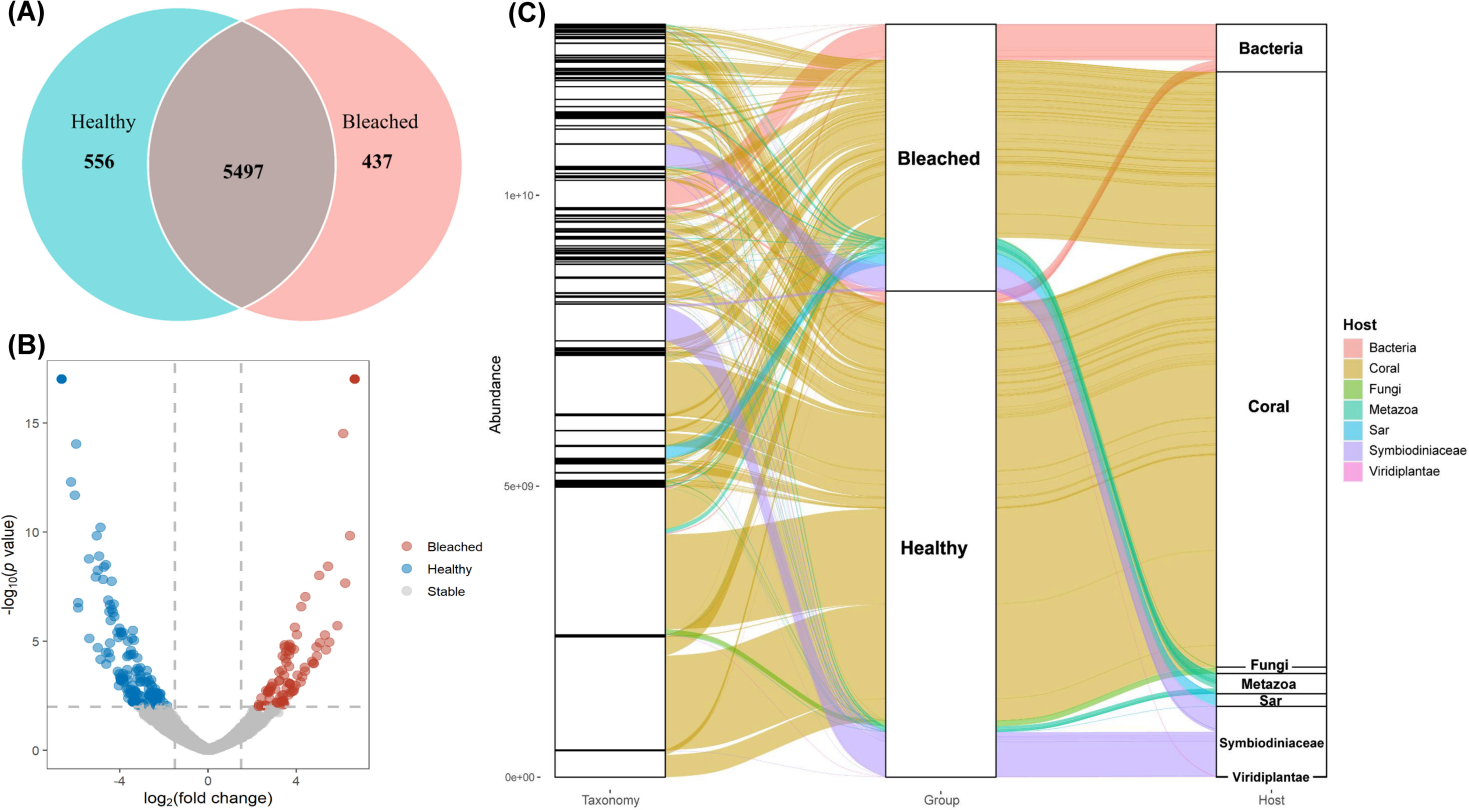
Metaproteome comparative analysis of healthy and bleached corals. (**A**) Venn diagram showing the number of proteins identified in healthy and bleached corals. (**B**) Volcano plot of differentially abundant proteins between healthy and bleached corals (*P* value < 0.01). (**C**) Sankey diagram showing the relationships between proteins, coral groups, and host sources of proteins.

To obtain a comprehensive perspective on the mechanisms underlying the physiological responses of coral symbionts to heat stress, we conducted a metaproteomic analysis of healthy and bleached corals that had suffered heat stress. Of the 6,490 proteins identified across all samples, 556 were unique to the healthy group, 437 were unique to the bleached group, and 5,497 were shared by the two groups (Fig. 4A). There were 410 differential proteins (log_2_(fold change)>1.5 and *P* < 0.05) in the healthy group, and 389 in the bleached group (Fig. 4B).

The overall abundance of differential proteins was higher in the healthy group than the bleached group (Fig. 4C). Corals were the most abundant host source, accounting for 85.95% of the total in the healthy group. The remaining differential proteins in the healthy group were primarily derived from Symbiodinium (9.17%), bacteria (2.45%), and metazoa (1.13%). In the bleached group, the majority of the differential proteins were derived from coral (66.47%), with the remaining proteins mainly being derived from bacteria (13.46%) and Symbiodinium (9.58%), and a small proportion being derived from metazoa (5.52%).

**Fig 5:**
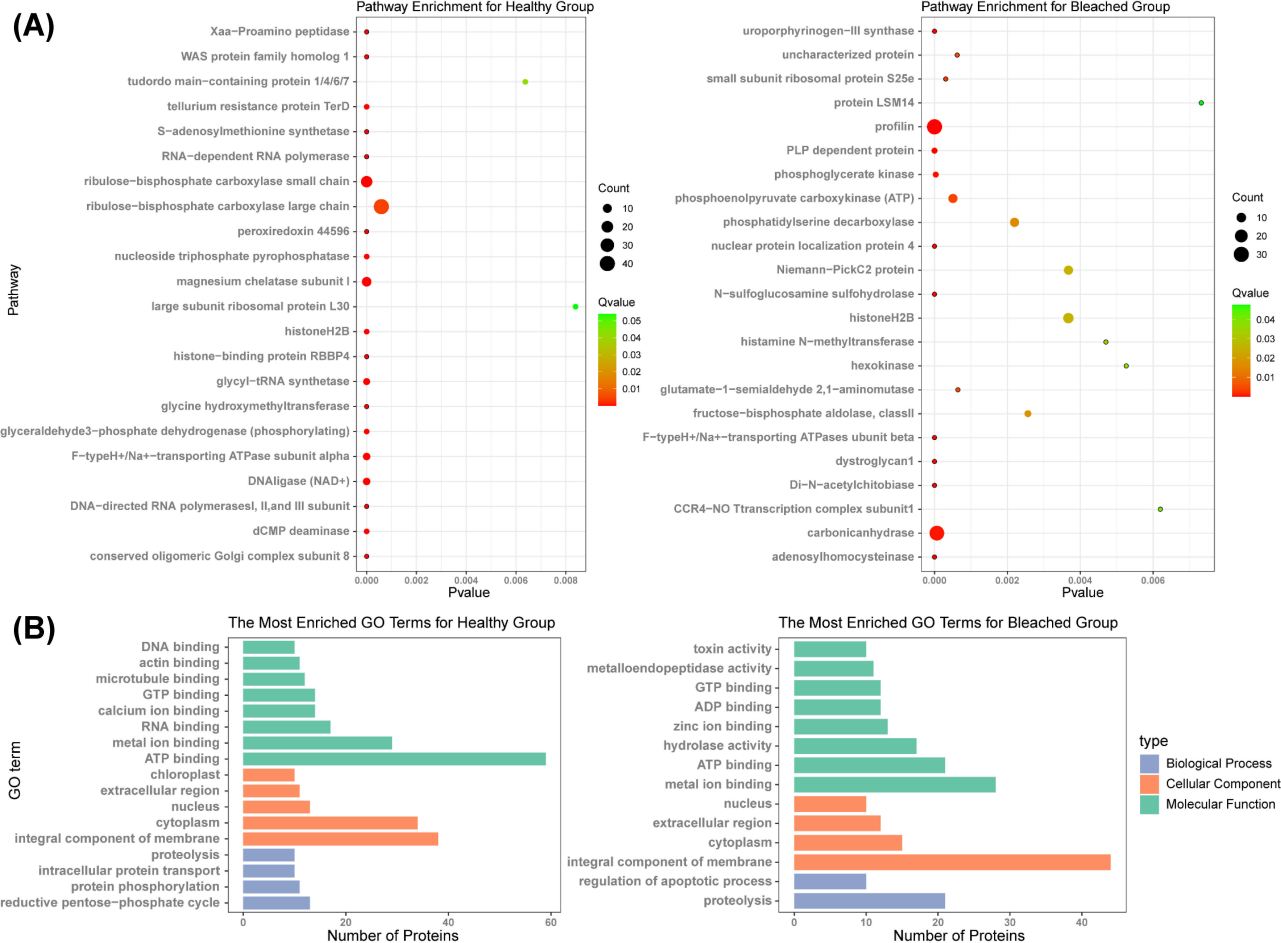
Functional analysis of the metaproteome of healthy and bleached coral symbionts. (**A**) Kyoto Encyclopedia of Genes and Genomes pathway enrichment analysis of significantly different proteins. (**B**) Gene Ontology (GO) term enrichment analysis of differentially expressed proteins.

To gain a better insight into the biological processes associated with the differential proteins, KEGG and Gene Ontology (GO) analyses were carried out. KEGG analysis revealed several enriched pathways in the healthy group, including pathways related to photosynthesis (such as ribulose-bisphosphate carboxylase), chlorophyll synthesis (such as magnesium chelatase subunit I), and sugar metabolism (such as glyceraldehyde-3-phosphate dehydrogenase) (Fig. 5A). KEGG analysis also indicated that profilin, phosphoenolpyruvate carboxykinase, phosphatidylserine decarboxylase, Niemann-PickC2 protein, histoneH2B, and carbonic anhydrase were enriched in the bleached group (Fig. 5A).

The differential proteins were also subjected to GO analysis. Molecular function (MF) was the most enriched category in both the healthy and bleached groups, followed by the cellular component (CC) and biological process (BP) categories (Fig. 5B). Regarding the MF category, in the healthy group, binding-related subcategories represented the largest population and had the highest degree of enrichment, while the top enriched MFs in the bleached group were metal and zinc ion binding, hydrolase activity, ATP/ADP/GTP binding, metalloendopeptidase activity, and toxin activity. Regarding the CC category, chloroplast, extracellular region, nucleus, cytoplasm, and integral component of membrane were significantly enriched in the healthy group, while nucleus, extracellular region, cytoplasm, and integral component of membrane were significantly enriched in the bleached group. Regarding the BP category, proteolysis, intracellular protein transport, protein phosphorylation, and reductive pentose-phosphate cycle were enriched in the healthy group, while the regulation of apoptotic process and proteolysis were primarily enriched in the bleached group.

### Differential bacterial proteins between the healthy and bleached corals

**Fig 6:**
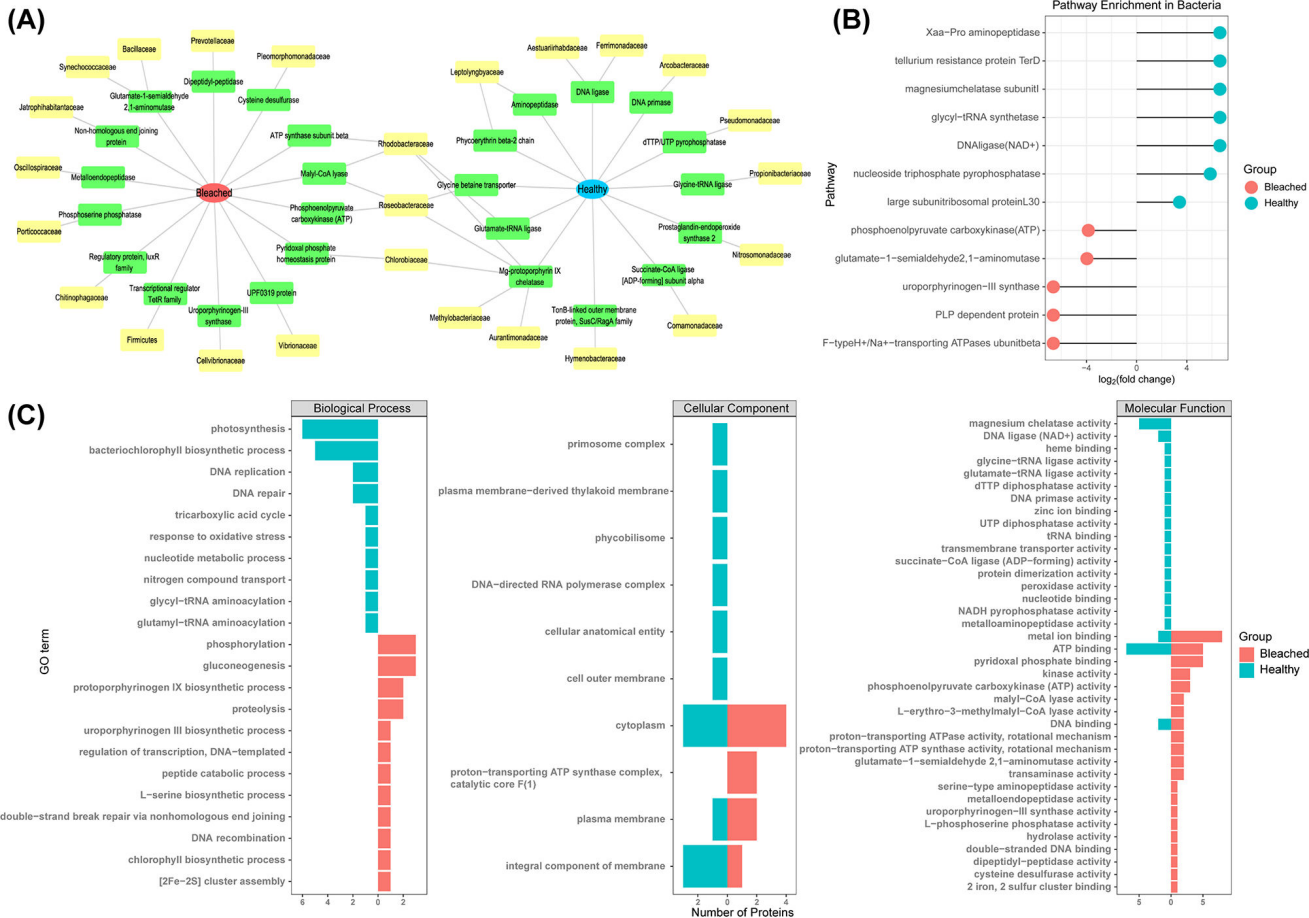
Metaproteome functional analysis of bacteria in corals. (**A**) Visual summary of differential functional proteins derived from bacteria. (**B**) Kyoto Encyclopedia of Genes and Genomes pathway enrichment analysis of significantly different proteins derived from bacteria in healthy and bleached corals. (**C**) Function of differently abundant proteins in bacterial response to heat stress in healthy versus bleached corals according to Gene Ontology terms.

To gain a deeper understanding of the functional disparities regarding bacterial proteins between the healthy and bleached groups, a comprehensive investigation was conducted on the differential proteins (between the healthy and bleached groups) derived from bacteria. The major bacterial families that significantly contributed to protein functions in the healthy group were Aurantimonadaceae, Arcobacteraceae, Comamonadaceae, Hymenobacteraceae, Leptolyngbyaceae, Methylobacteriaceae, Nitrosomonadaceae, and Pseudomonadaceae. In contrast, those in the bleached group were Bacillaceae, Firmicutes, Prevotellaceae, Vibrionaceae, Oscillospiraceae, and Synechococcaceae. Furthermore, Chlorobiaceae, Roseobacteraceae, and Rhodobacteraceae contributed in both groups (Fig. 6A).

Using KEGG and GO analyses of the differential bacterial proteins, we further explored the molecular mechanisms of coral bleaching related to bacteria. The top KEGG pathways enriched in the healthy group were related to Xaa-Pro aminopeptidase, tellurium resistance protein TerD, magnesium chelatase subunit I, glycyl-tRNA synthetase, DNA ligase, nucleoside triphosphate pyrophosphatase, and large subunit ribosomal protein L30 (Fig. 6B). On the other hand, the top KEGG pathways enriched in the bleached group were phosphoenolpyruvate carboxykinase, glutamate-1-semialdehyde-2,1-aminomutase, uroporphyrinogen III synthase, pyridoxine phosphate (PLP)-dependent protein, and F-type H^+^/Na^+^-transporting ATPases subunit beta.

Various patterns and molecular mechanisms were identified based on the annotated GO terms for bacterial proteins. Regarding the BP category, the main enriched terms in the healthy group were photosynthesis, bacteriochlorophyll biosynthetic process, DNA replication and repair, tricarboxylic acid cycle, response to oxidative stress, nucleotide metabolic process, nitrogen compound transport, and glycyl-tRNA aminoacylation (Fig. 6C). However, those in the bleached group were phosphorylation, gluconeogenesis, protoporphyrinogen IX biosynthetic process, proteolysis, uroporphyrinogen III biosynthetic process, regulation of transcription, peptide catabolic process, L-serine biosynthetic process, double-strand break repair via non-homologous end joining, DNA recombination, chlorophyll biosynthetic process, and [2Fe-2S] cluster assembly (Fig. 6C).

Regarding the CC category, both groups exhibited enrichment for functions related to membrane, such as the integral component of membrane and plasma membrane. Additionally, differential proteins associated with the proton-transporting ATP synthase complex were enriched in the bleached group. In contrast, the healthy group exhibited enrichment in the primosome complex, plasma membrane-derived thylakoid membrane, phycobilisome, DNA-directed RNA polymerase complex, cellular anatomical entity, and cell outer membrane.

Regarding the MF category, the healthy group showed significant enrichment in activity-related functions, such as magnesium chelatase activity, ligase activity, dTTP diphosphatase activity, DNA primase activity, UTP diphosphatase activity, transmembrane transporter activity, succinate-CoA ligase activity, protein dimerization activity, peroxidase activity, NADH pyrophosphatase activity, and metalloaminopeptidase activity. Similarly, most of the major differential proteins enriched in the bleached group had activity-related functions, such as kinase activity, phosphoenolpyruvate carboxykinase activity, malyl-CoA lyase activity, aminomutase activity, transaminase activity, serine-type aminopeptidase activity, metalloendopeptidase activity, phosphatase activity, hydrolase activity, and cysteine desulfurase activity. In addition, differential proteins involved in binding function, including heme binding, tRNA binding, ATP binding, DNA binding, nucleotide binding, metal ion binding, and pyridoxal phosphate binding, were found in the healthy and bleached groups.

## DISCUSSION

### Bleaching significantly altered the community composition and function of coral symbionts

Individual responses of coral symbionts to heat stress are critical for coral acclimation and survival under climate change (23, 24). This study compared the symbiont community composition and functional differences of healthy and bleached corals (*Acropora millepora*) that suffered heat stress using metagenomic and metaproteomic analyses. Our findings identified many distinct and adaptive traits in the healthy group, as well as intricate shifts in both function and taxonomic structure related to bleaching processes in the bleached group.

Emerging evidence suggests that diverse bacteria are associated with coral and that the bacterial communities are linked to the thermotolerance of their hosts (25). Severe disruption of the natural microbial community by external stresses can irreversibly affect the fate of the coral symbionts. In particular, potential opportunistic pathogens dramatically increase in bleached corals, causing immune responses and unstable states in corals, which can cause algal symbiont elimination and coral diseases (19, 26). In this study, there were large differences in community structure between the healthy and bleached groups after thermal bleaching. The total number of species was notably higher in the bleached tissues, where bacteria predominated, compared to the healthy group (Fig. 1). The relative abundances of several bacteria such as Vibrionaceae, Rhodobacteraceae, Chlorobiaceae, and Sphingomonadaceae were increased in the bleached group compared to the healthy group, while Oxalobacteraceae and Moraxellaceae were decreased (Fig. 1D). Various *Vibrio* species belonging to the family Vibrionaceae often act as conditional pathogens and play a triggering role in coral disease (27). Several members of the Rhodobacteriaceae family are indicator species of thermal stress and predominate in corals exposed to elevated seawater temperature (28). Furthermore, Chlorobiaceae (green bacteria) and Sphingomonadaceae (alpha-proteobacteria) usually adopt the r-strategy (29), which has been described by Cushing (30) as a kind of opportunism that involves maximizing reproduction and occurs in unstable environments, in contrast to the K-strategy, which is a strategy that occurs in stable environments. Bleaching represents a disturbance, which may help to account for the increases in Chlorobiaceae and Sphingomonadaceae. In comparison, Oxalobacteraceae is a key indicator of healthy mucus microbiomes (31), which favors coral health. Furthermore, some members of the Moraxellaceae family can degrade organic matter and may use this ability to recycle carbon for corals (32). The observed decrease in the relative abundance of beneficial bacteria in the bleached group demonstrates that the loss of beneficial microbiome members may provide niches for other bacteria and pose a serious health threat to coral symbionts, often associated with an increase in opportunistic pathogens (33). Our results indicate that bleaching disrupts the ratio of beneficial to harmful bacteria in coral symbiont communities and that stabilizing the coral microbial composition could serve as an ecological strategy to buffer against heat stress.

In addition, we observed similar symbiont community composition in the three parallel samples in the healthy group, whereas there were significant differences in the three parallel samples in the bleached individuals. This phenomenon concurs with the Anna Karenina Principle (AKP) in the microbial field (34, 35), which states that “all healthy microbiota are alike; each disease-associated microbiota is sick in its own way.” Symbiont dysbiosis can be conceptualized in terms of the AKP as a temporary loss of the host capacity to regulate the host microbiota, implying that loss of homeostasis decreases host fitness.

In addition, microbes play crucial roles in nutrient cycling in coral symbiont communities and thus may mediate symbiont responses to environmental stresses (36). Analysis of functional differences between the healthy and bleached groups after heat stress can offer insights into the mechanisms associated with bleaching. Our metagenomic analysis, which involved KEGG and COG annotation, revealed that metabolism processes, including carbon, carbohydrate, fatty acid, amino acid, and microbial metabolism, were significantly enriched in the bleached group (Fig. 2). Conversely, oxidative phosphorylation (energy metabolism subcategory) and thermogenesis (environmental adaptation subcategory) were dramatically enriched in the healthy group. The coral hosts were the main contributors to the functional genes in the healthy group, whereas in the bleached group, the bacteria Vibrionaceae, Rhodobacteraceae, and Chlorobiaceae replaced the host as the main functional contributors (Fig. 3).

Mounting evidence has revealed the role of nutritional mechanisms in coral responses to heat stress and bleaching processes (37, 38). Under thermal stress, the relative contribution of symbionts to carbon metabolism decreases due to the breakdown of the coral-algae endosymbiosis and decrease in photosynthetic products (39). Meanwhile, impaired photosynthesis affects fatty acid metabolism, resulting in reduced lipid production and altered fatty acid composition. Coral hosts are forced to catabolize stored carbon reserves to maintain the energy required for symbiotic photosynthesis. Once the resource is depleted, photosynthetic dysfunction ensues, leading to coral bleaching via the photo-oxidative pathway (40). Furthermore, bleached corals respond to lower endosymbiont concentrations by catabolizing lipids or maintaining energy reserves as their bleaching recovery strategy (41). The healthy group maintained symbiont survival as a result of lower metabolism and energy consumption. In contrast, although the significant metabolic enrichment in bleached coral symbionts may represent a method of coral bleaching recovery, metabolic abnormalities provide opportunities for potentially pathogenic bacteria, such as *Vibrio*, to disrupt fragile symbiotic systems. Increases in these pathogens may deplete nutrients in corals and deteriorate the micro-environment, ultimately leading to irreversible coral bleaching.

The ability of symbiotic bacteria to metabolize specific carbohydrates enables them to adapt to the substrate niche of the hosts. This makes the carbohydrate composition a key factor in shaping the microbial community (42). Exudates secreted by coral symbionts increase the growth of specific bacterial species and maintain the growth of other specific bacterial species (43). When coral bleaching occurs, changes in the chemical composition of the coral mucus can alter the bacterial communities and functions (44, 45). We found that the healthy group predominantly harbored glycosyl transferase genes, while the bleached group contained a higher abundance of glycoside hydrolase genes (Fig. 2C). In addition, the carbohydrate-active enzyme (CAZy) genes were found to be derived from the coral host in the healthy group, while they were mainly derived from Vibrionaceae, Rhodobacteraceae, and Chlorobiaceae in the bleached group (Fig. 3C). Many families of glycoside hydrolases target the glycosidic linkages of N-acetylmuramoyl or N-acetylglucosaminyl residues in peptidoglycan, which accounts for a large proportion of the chemical composition of coral mucus (46). Given the high abundance of glycoside hydrolase genes in the bacterial communities in the bleached group, these bacteria may have the ability to N-glycosylate coral mucus and penetrate the host mucus layer in order to establish endosymbiosis (47). Therefore, potential pathogens may be able to penetrate the coral mucus or surface biofilm using hydrolytic enzymes during coral bleaching, breaking through the coral’s first line of defense against invasion and inducing diseases.

Meanwhile, bacterial pathogens utilize certain genes, such as virulence factors, to interact with the host and cause damage or disease (48). It was notable that virulence factor genes were considerably enriched in the bleached group, with the majority of these genes being derived from Vibrionaceae (Fig. 2D and 3D). During coral bleaching, pathogens can break through the mucus layer to enter host cells and interact with the host via multiple mechanisms, including by using virulence factors related to iron and magnesium uptake systems, adherence, secretion systems, toxins, stress proteins, and invasion. Adhesion to coral surfaces is the key to invasion by pathogenic bacteria, while the iron uptake system is a non-specific virulence system related to competition skills (49). Additionally, the secretion systems and toxin production of pathogens play significant roles in host damage and competition with the commensal microbiome during colonization. For example, virulence factors released by *Vibrio* can cause changes in the coral microbiome that favor the pathogenic potential of the entire *Vibrio* community (50, 51). Together with previous findings (26), our findings suggest that heat stress can lead to an increased threat from pathogens among corals, providing a possible explanation for the high prevalence of many coral diseases in the context of global warming.

### Bleaching disrupts the metabolic potential of coral symbionts at the protein level

The response of coral symbionts in the bleached group was pronounced at the genetic level. To obtain evidence at the protein level, a metaproteomic analysis was performed to determine the differential proteins between the healthy and bleached groups. We identified many differential proteins, which may be associated with the differences in bacterial communities between the two groups (Fig. 4B). Although the differential proteins were mainly derived from the coral host in both groups, the abundance of bacteria-derived differential proteins was dramatically higher in the bleached group than in the healthy group (Fig. 4C).

The healthy group exhibited enrichment in a range of differential proteins related to photosynthesis and chlorophyll biosynthesis (such as ribulose-bisphosphate carboxylase and magnesium chelatase) (Fig. 5A). Zooxanthellae fix CO_2_ directly via ribulose-bisphosphate carboxylase/oxygenase (52). Corals maintain their maximum photosynthetic capacity by ensuring that the equilibrium between the carboxylase and oxygenase activity of this enzyme favors carbon fixation (53). High temperatures can trigger the onset of CO_2_-limited conditions around the enzyme, leading to the breakdown of the coral-algae symbiosis (39). Chlorophyll biosynthesis involves ATP-dependent insertion of a magnesium ion into protoporphyrin IX, catalyzed by three magnesium chelatase subunits (54). Increased chlorophyll synthesis in symbionts alters the pigment composition to fit the external conditions (55). The significant enrichment of these proteins in the healthy group suggests that the normal function of the symbionts is maintained during bleaching by maximizing photosynthesis and chlorophyll synthesis.

Bleaching is the destructive endpoint of a suite of cellular processes, and the differential proteins enriched in the bleached group after exposure to high temperatures were associated with host skeletal function (including profilin and carbonic anhydrase) and proteins related to gluconeogenesis (phosphoenolpyruvate carboxykinase) (Fig. 5A). Profilin is an actin-binding protein involved in the dynamic turnover and restructuring of the actin cytoskeleton (56). Carbonic anhydrase plays an important role in coral calcification and is involved in the biomineralization of many scleractinian corals (57). Phosphoenolpyruvate carboxykinase upregulation indicates that coral hosts compensate for the loss of symbiont-derived nutritional products by converting their internal energy stores into carbohydrates (58). Therefore, our study indicates that in the event of a coral-algae system collapse, the remaining symbionts maintain host skeletal calcification and perform energy conversion in an attempt to compensate for the loss of zooxanthellae.

Further, GO analysis indicated that healthy corals responded to heat stress by altering their carbon metabolism, exhibiting significant enrichment in reductive pentose-phosphate cycle proteins (Fig. 5B). The pentose-phosphate pathway is involved in primary carbon metabolism closely interconnected with glycolysis and plays an important role during oxidative stress and antistress responses (59). In addition, protein phosphorylation, a mechanism for regulating and controlling protein activity and function, was significantly enriched in the healthy group. Previous research has shown that decreased protein phosphorylation triggered temperature-induced coral bleaching (60). In contrast, the bleached group was enriched in proteins associated with toxin activity and hydrolase activity. Toxin activity refers to causing injury to other living organisms, usually associated with pathogens. Metalloendopeptidases, which rely on metal ions such as zinc ions for their catalytic activity, are known toxic factors in a wide range of bacterial pathogens (61). Therefore, these results indicate that expression and release of toxins by pathogens were affected by high seawater temperature. Moreover, upregulation of genes related to hydrolase activity and proteolysis has been shown to be associated with thermal stress, contributing to increased catabolism of carbohydrates and amino acids in corals (62). Additionally, proteins associated with the regulation of apoptotic processes were enriched in the bleached group. Heat stress can induce apoptotic processes in the host, which may lead to coral bleaching and even mortality (63). For algal symbionts, apoptosis is one of the cellular processes involved in zooxanthellae elimination (64). Activation of antiapoptotic molecules in host cells can serve as an important mechanism for regulating the apoptotic response, highlighting the attempt by corals to rapidly inhibit apoptosis to ensure thermotolerance and survival (65).

### Driving role of bacterial micro-ecological profiles in coral bleaching

Both the metagenomic and metaproteomic data in this study demonstrated remarkable increases in the relative abundance of bacteria and functional bacterial genes in the bleached group, indicating their critical role in coral bleaching. Given the prominence of bacteria among the symbionts, detailed functional analyses of the bacteria were performed (Fig. 6). Several bacteria with nutrient cycling functions, such as Comamonadaceae, Methylobacteriaceae, and Nitrosomonadaceae, play essential roles in the health of corals. Comamonadaceae are denitrifying bacteria that have been found in activated sludge (66) and were shown to be involved in the energy transfer process in our study. Several Methylobacteriaceae have been identified as nitrogen-fixing symbionts (67) and were shown to be associated with chlorophyll biosynthesis in our study. Nitrosomonadaceae are lithoautotrophic ammonia oxidizers that are known to control the nitrogen cycle in terrestrial, freshwater, and marine environments (68). In comparison, bacteria such as Vibrionaceae, Cellvibrionaceae, and Synechococcaceae were associated with biological processes in the bleached group such as metalloendopeptidases related to toxicity, and cysteine desulfurase linked to sulfur-related biosynthetic pathways. These findings suggest a complex interplay between different bacterial families and their functions during coral bleaching.

Functional annotation of differential proteins derived from bacteria revealed the critical role of bacteria in maintaining basic symbiont functions and resisting stress in healthy corals (Fig. 6B). Specifically, bacteria in the healthy group were found to perform vital cellular functions, including catalyzing the synthesis of glycyl-tRNA (glycyl-tRNA synthetase) and DNA repair (DNA ligase), along with being involved in chlorophyll synthesis (magnesium chelatase) and bacterial resistance to heavy metals (tellurium resistance protein TerD (69). These results were further supported by the GO BP analysis (Fig. 6C), which showed that bacteria in the healthy group were primarily involved in photosynthesis and the biosynthesis of bacteriochlorophyll. The presence of bacteriochlorophyll may indicate that aerobic anoxygenic phototrophic bacteria have a photosynthetic apparatus capable of utilizing sulfide as an electron donor (70). In addition, the bacteria in the healthy group were involved in DNA repair and responses to oxidative stress. A common mechanism underlying the stress response of corals to high temperature is oxidative stress, which is exacerbated by high solar irradiance accompanied by thermal insulation (64). Oxidative stress can indirectly cause DNA damage, leading to apoptosis or programmed cell death if not repaired. The previous study showed that coral symbionts had a robust antioxidant response when exposed to heat stress (71). In summary, the results of this study suggest that bacteria in the healthy group performed an alternative photosynthesis function after the corals experienced heat stress while exhibiting antioxidative stress responses and participating in DNA repair processes to repair DNA damage. This may be crucial for coral symbionts’ recovery from initial bleaching processes.

Conversely, in the bleached group, which involved corals that underwent heat stress without recovery, bacteria were involved in energy metabolism and catalytic activity. Phosphoenolpyruvate carboxykinase and PLP-dependent enzyme were enriched in bacteria in the bleached group. The former enzyme is linked to gluconeogenesis, which was also found to be enriched in our GO BP analysis (Fig. 6C). A previous analysis of gene expression in corals under heat stress found that this enzyme was significantly upregulated only in bleached corals, and it may be the rate-limiting regulatory step in the chain of events from lipid breakdown to carbohydrate synthesis (58). In addition, the latter enzyme has catalytic activity, which has been found to be involved in stress responses in corals (72). Further, we also found that the bacteria in the bleached group were involved in various biosynthetic processes such as the biosynthesis of protoporphyrinogen IX, uroporphyrinogen III, and chlorophyll, which are all associated with chlorophyll synthesis. Bacteria capable of chlorophyll biosynthesis (including Firmicutes and Synechococcaceae) were present in the bleached group. A previous study reported that five phyla (Firmicutes, Cyanobacteria, Proteobacteria, Chlorobi, and Chloroflexi) can convert light energy into chemical energy using a photochemical reaction center (73). Clearly, some of the bacteria in the bleached group converted light energy into internal energy for the symbionts to survive after experiencing heat stress, to compensate for the loss of zooxanthellae. Moreover, proteolysis and peptide catabolic process were also enriched in bacteria in the bleached group. As mentioned above and in previous research (74), this may be related to the catabolism of amino acids in corals, as well as the proliferation of pathogens and the occurrence of diseases associated with thermal bleaching. Taken together, these findings indicate that the regulation of coral symbionts is diminished when they encounter stress, which allows for pathogen colonization and the release of virulence factors, ultimately resulting in host metabolic abnormalities and eventually bleached. Future precise studies of the genetic and functional characterization of bacteria will facilitate a better understanding of their roles in coral symbiont communities experiencing heat stress.

### Conclusions

This study investigated the post-heat stress differences in the gene and protein abundances and functions between healthy and bleached corals. Metagenomic analysis revealed that bleaching was associated with alterations in the coral symbiont community, with increases in potential pathogens and virulence genes. Metaproteomic analysis confirmed the impact of heat stress on the metabolism and basic functions of coral symbionts, and highlighted the role of bacteria in mediating metabolic functions that affect the adaptability and resilience of the coral symbiont community. Overall, the study revealed differences in energy supply strategies between healthy and bleached corals. In response to the disruption of the symbiotic system by heat stress and reduced carbohydrate supply, coral symbionts maintain their own energy requirements by altering energy utilization in order to gradually restore coral host health. The proliferation of opportunistic pathogens in corals can lead to host metabolic abnormalities and energy disturbances, which can lead to further bleaching and even coral death. Overall, the community composition and functions of coral-associated bacteria were strongly related to the occurrence of bleaching, providing evidence for our hypothesis that bleaching may be related to bacterial micro-ecological profiles. This study elucidates how thermal stress affects the composition and functions of coral symbionts, which improves our understanding of coral acclimatization and resilience to thermal bleaching.

## MATERIALS AND METHODS

### Study site and coral sampling

In September 2020, coral samples were collected from the Luhuitou fringing reef (18°13′N, 109°28′E), which is situated near Sanya City, Hainan Island, on the south coast of China (Fig. S1). A severe coral bleaching event occurred in the summer of 2020, which was caused by record-breaking warm sea surface temperature anomalies in the South China Sea. The bleaching was widespread across the fringing reef along the Chinese mainland and around Hainan Island (22). The mean annual sea surface temperature was 27°C, and the mean monthly temperature ranged from 23.1°C to 29.8°C, while the seawater temperature during sample collection was 30.2°C ± 0.2°C. In addition, during the sampling process, the salinity was 32.1% ± 0.3‰ and the pH was 8.13 ± 0.15, which were detected by multi-parameter water quality automatic monitoring sensor (Bescient Technologies, Shenzhen, China).

Six colonies of *Acropora muricata* were haphazardly sampled at a depth of 4–6 m, with three visually healthy colonies and three bleached colonies being selected. To minimize the potential impact of coral age on microbiota composition, adult coral colonies of equivalent size (approximately 20–30 cm in diameter) were haphazardly selected. Independent specimens (~10 to 20 cm^2^) were obtained from each colony using a hammer and chisel while scuba diving, with a sampling interval distance of ≥5 m. To remove the microbes attached to the surface, the coral samples were rinsed with sterilized seawater. Each specimen was then split into two fragments, one for metagenomic sequencing and the other for metaproteomic analysis. All samples were individually sealed in ziplock bags and immediately kept on dry ice until storage at −80°C before further processing.

### DNA extraction and metagenomic sequencing

The coral samples were immersed in liquid nitrogen and ground for 30 s at 60 Hz using a pre-cold tissue grinder (Tissuelyser; JingXin, Shanghai, China). The total DNA from healthy and bleached tissue slurries was separately extracted using a MoBio PowerSoil DNA Isolation Kit (MoBio Laboratories, Carlsbad, CA, USA) according to the manufacturer’s instructions. Agarose gel electrophoresis was used to validate the integrity and purity of the extracted DNA. The concentration and quality of the DNA were assessed using a Quantus Fluorometer (Promega, Madison, WI, USA) and a NanoDrop 2000 Spectrophotometer (Thermo Fisher Scientific, MA, USA), respectively. An optical density OD_260_/OD_280_ of > 1.8 was used to indicate DNA quality.

DNA libraries were prepared with NEXTFLEX Rapid DNA-Seq (Bioo Scientific, Austin, TX, USA) and sequenced using a paired-end configuration with an Illumina HiSeq platform (Illumina Inc., San Diego, CA, USA) following the manufacturer’s standard protocols. Image analysis and base calling were carried out using MiSeq Control Software. The sequencing runs and data filtering were conducted by Majorbio, Inc. (Shanghai, China).

### Metagenomic data analysis

Raw sequences initially underwent adaptor trimming and then reads <50 bp, reads containing N bases, or reads with a quality score of <20 were discarded using fastp (75). High-quality reads were then assembled into contigs using MEGAHIT (76). Contigs ≥300 bp were used for subsequent gene prediction and annotation. Open reading frames from each assembled contig were predicted using MetaGene (77) and translated into amino acid sequences using National Center for Biotechnology Information translation tables. Subsequently, a non-redundant gene catalog was constructed using CD-HIT (78) with 90% identity and coverage. High-quality reads were aligned to this catalog, and gene abundances based on >95% identity were calculated using SOAPaligner (79).

Based on the Non-Redundant Protein Sequence Database, KEGG database, and COG database, the taxonomy, metabolic function, and protein orthologous groups of representative sequences, respectively, were annotated using Diamond (80). CAZy annotation was performed using hmmscan against the CAZy database, and virulence factor annotation was conducted based on the VFDB using Diamond (80). Subsequent statistics were analyzed using the online Majorbio Cloud Platform (www.majorbio.com).

### Protein extraction and metaproteomic sequencing

Coral tissue (10 g) was subjected to centrifugation at 4,000 *g* for 5 min in order to collect the mucus, followed by centrifugation at 20,000 *g* for 20 min to remove impurities. The resulting supernatant was precipitated overnight at −20°C with 4 vol of acetone. The proteins were extracted by centrifugation at 20,000 *g* and 4°C for 10 min. After centrifugation at 20,000 *g* and 4°C for 20 min, the sediment was dissolved in 5× sodium dodecyl sulfate sample buffer for mass spectrometry (MS) analysis.

To perform protein fractionation, 10-µL samples were loaded onto a homemade 10% sodium dodecyl sulfate-polyacrylamide gel electrophoresis gel and separated under reducing conditions. The gel was stained using a Simple Blue kit (Thermo Fisher Scientific). Next, each sample was then subjected to in-gel reduction, alkylation, and tryptic digestion. In brief, the proteins were reduced using 10-mM Tris(2-carboxyethyl)-phosphine for 1 h, alkylated using 50-mM iodoacetamide for 1 h at room temperature, and then digested using sequence-grade, modified trypsin 1:20 (wt/wt) (Promega) overnight at 37°C in 50-mM NH_4_HCO_3_ (pH 8.0). The reaction was quenched by acidification with 1% formic acid (Thermo Fisher Scientific). The resulting peptide mixture was desalted using reverse phase C18 Stop and Go Extraction Tips (Thermo Fisher Scientific) and diluted in 0.1% formic acid prior to nanohigh-performance liquid chromatography and mass spectrometry (HPLC-MS) analysis.

A mass spectrum was conducted using an EASY-nLC 1200 system (Thermo Fisher Scientific) connected to an Orbitrap Eclipse mass spectrometer (Thermo Fisher Scientific). In brief, tryptic peptides (700 ng in 0.1% formic acid) were loaded onto a nano-trap column (75-µm i.d.  × 2 cm precolumn, Acclaim PepMap 100 C18, 3 µm, 100 Å; Thermo Fisher Scientific). Subsequent separation was performed on an analytical column (50 µm  ×  15 cm, Acclaim PepMap RSLC C18, 2 µm, 100 Å; Thermo Fisher Scientific) using an initial gradient ranging from 2% to 8% of buffer B (80% acetonitrile and 0.1% formic acid) for 5 min, followed by a second gradient ranging from 8% to 43% of buffer B for 80 min, and a third gradient ranging from 43% to 50% of buffer B for 5 min at a flow rate of 300 nL/min. The mass spectrometer was operated in the data-dependent mode for acquisition. Survey scans were recorded in the Orbitrap at 60,000 resolution (automatic gain control 4e5, max injection time 50 ms), with a scan range from 350 to 1,600 *m*/*z*. MS/MS scans were recorded in the Orbitrap at 30,000 resolution (automatic gain control 1e^5^, max injection time 120 ms, isolation width 1.6 *m*/*z*). Unknown, singly charged, and doubly charged ions were excluded from fragmentation. Selected precursors were fragmented by applying a higher energy collisional dissociation energy of 30% normalized collision energy.

### Metaproteomic data analysis

Analysis and identification of peptides were conducted using Proteome Discoverer version 2.5 (Thermo Fisher Scientific) with the following parameters: a maximum of three missed cleavage sites for trypsin per peptide, cysteine iodoacetamide methylation as a fixed modification, and methionine oxidation as a dynamic modification. Search results were filtered based on precursor tolerance (±10 ppm) and fragment tolerance (±20 ppm). Protein taxonomic assignments were conducted by the Mascot algorithm (Matrix Science, London, UK) using Proteome Discoverer version 2.5, searching against an amino acid sequence database that was based on the *A. muricata* (coral) metagenome. Classification of each functional protein was determined using the UniProt GO annotation program (www.uniprot.org).

### Statistical analysis

Differentially abundant genes between the metagenomes of the healthy and bleached groups were identified by two-tailed *t*-tests, with an adjusted *P* < 0.05 representing a significant difference. Differentially expressed proteins between the healthy and bleached groups were identified by *t*-tests, with *P* ≤ 0.01 and log_2_(fold change) greater than 1.5 or less than –1.5 representing significantly different proteins. Subsequently, the proteomes were analyzed using a volcano plot of −log_10_(*P* value) vs log_2_(fold change). Significant correlations of differential proteins between the healthy and bleached groups were calculated using the correlation analysis, and the results were visualized using the “ggplot2” (81) and “ggpubr” (82) packages in R (83).

## Data Availability

The metagenomic raw sequences generated in this study have been deposited in the National Center for Biotechnology Information Sequence Read Archive database (accession number PRJNA954896). The mass spectrometry proteomic data have been deposited with the ProteomeXchange Consortium (http://proteomecentral.proteomexchange.org) via the iProX partner repository (84, 85) (data set identifier PXD041598).

## SUPPLEMENTAL MATERIAL

Supplemental figures msystems.00505-23-s0001.pdf Fig. S1 and S2.

ASM does not own the copyrights to Supplemental Material that may be linked to, or accessed through, an article. The authors have granted ASM a non-exclusive, world-wide license to publish the Supplemental Material files. Please contact the corresponding author directly for reuse.
