# Supplementary material for: Integrated metagenomic and metaproteomic analyses reveal bacterial micro-ecological mechanisms in coral bleaching
Source: mSystems. 2023 Oct 26;8(6):e00505-23. doi: 10.1128/msystems.00505-23 (PMC10734480; doi:10.1128/msystems.00505-23)
Supplement: Supplemental figures — Fig. S1 and S2. [file msystems.00505-23-s0001.pdf]

Integrated metagenomic and metaproteomic analyses reveal bacterial micro-ecological mechanisms in coral bleaching

Keke Cheng[a], Xinyang Li[a], Mengmeng Tong[b], Mui-Choo Jong[c], Zhonghua Cai[a], Huina Zheng[d], Baohua Xiao[d**], Jin Zhou[a*]

[a]Shenzhen Public Platform for Screening and Application of Marine Microbial Resources, Shenzhen International Graduate School, Tsinghua University, Shenzhen 518055, PR China.

[b]Ocean College, Zhejiang University, Zhoushan 316021, PR China.

[c]Institute of Environment and Ecology, Shenzhen International Graduate School, Tsinghua University, Shenzhen 518055, PR China.

[d]Shenzhen Institute of Guangdong Ocean University, Shenzhen 518114, PR China.

*Corresponding author: Jin Zhou

Address: Room 902, Marine-Building, Shenzhen International Graduate School, Tsinghua University, Xili University Town, Shenzhen city, 518055, Guangdong Province, PR China.

Tel: +86-755-86953413; Fax: +86-755-86953413

E-mail address: zhou.jin@sz.tsinghua.edu.cn

** Co-corresponding author, Baohua Xiao, E-mail addresses: xiaobh@gdou.edu.cn

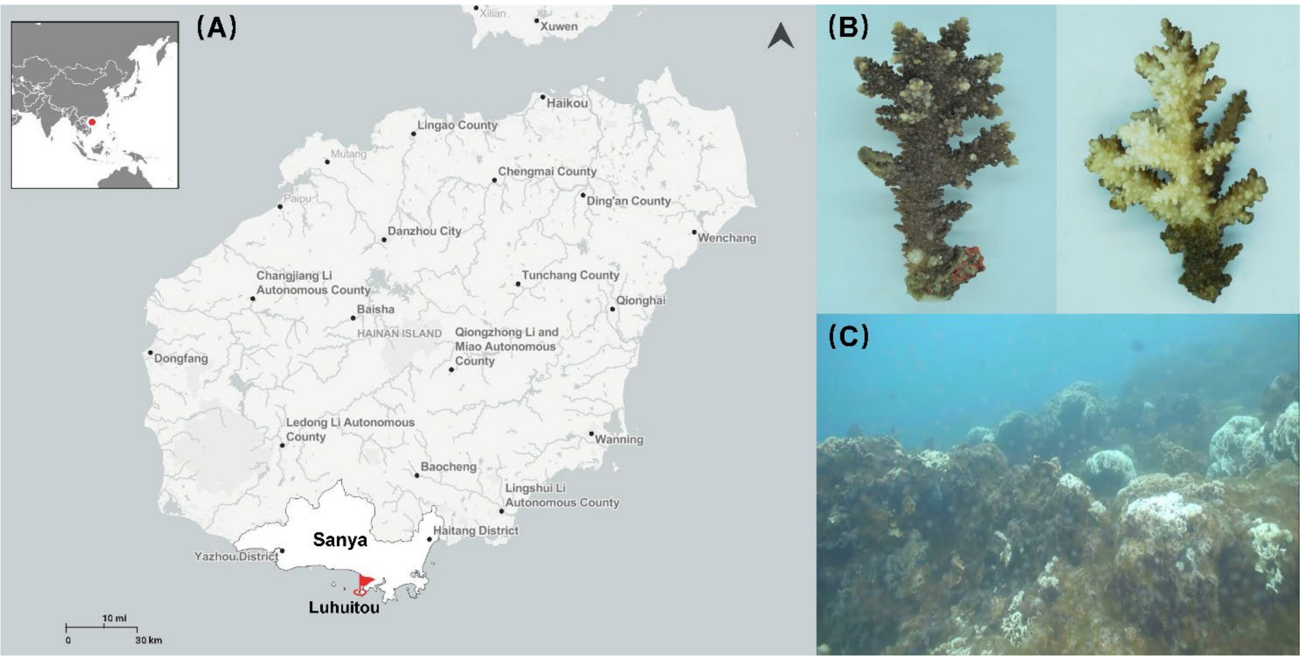

FIG S1 (A) A geographic map of the coral sampling site on the Luhuitou fringing reef in Sanya City, Hainan Island, on the south coast of China. (B) Representative real-time captured image of healthy and bleached target corals. (C) In situ monitoring of coral bleaching through fixed undersea observation equipment at the seabed.

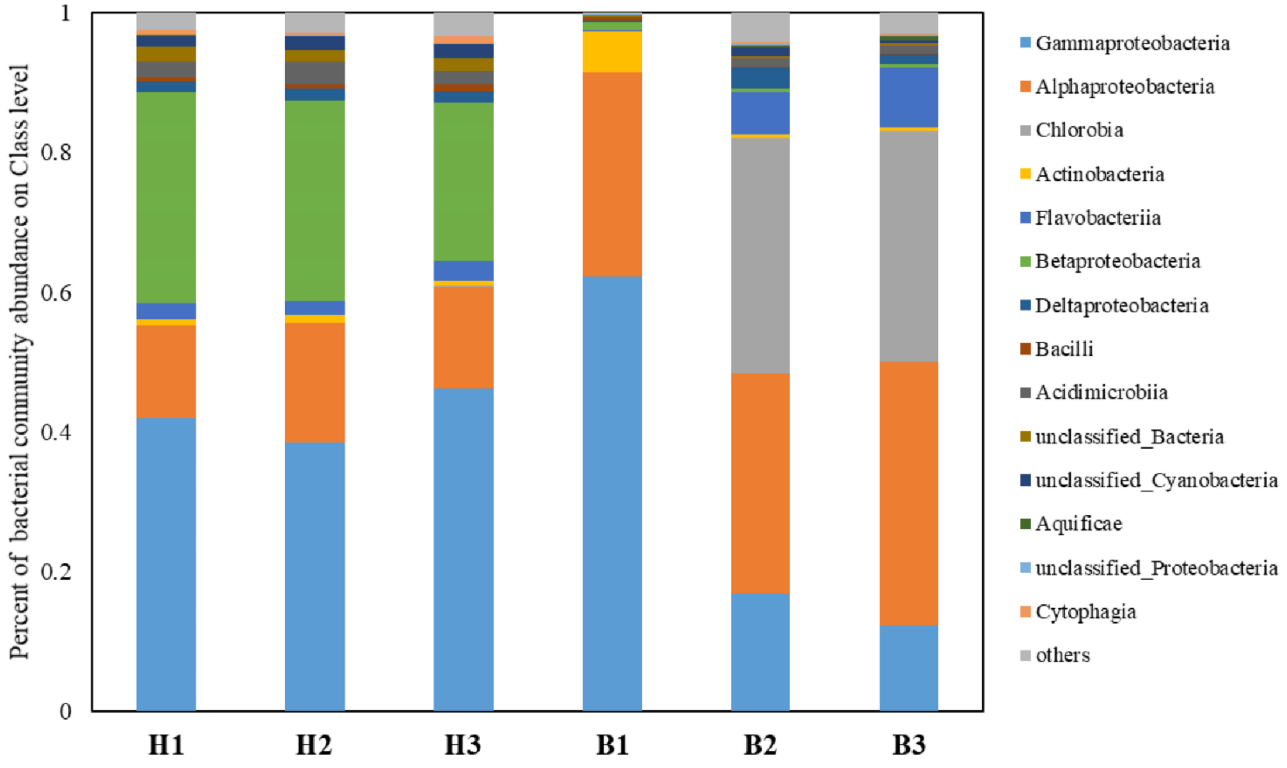

FIG S2 Relative abundances of bacterial community at the class-level in each coral individuals.
